# Can culture and universalism co-exist in affective neuroscience?

**DOI:** 10.3389/fpsyg.2026.1797537

**Published:** 2026-05-08

**Authors:** Gökçe Özkarar-Gradwohl, Oliver Hugh Turnbull

**Affiliations:** 1 Çinar Counseling, Istanbul, Türkiye; 2 School of Psychology, Bangor University, Bangor, Wales, United Kingdom

**Keywords:** affective neuroscience, cross-cultural affective neuroscience, culture, emotion, personality, universalism

## Introduction

In the last few decades, data on the brain basis of basic emotions have led to a new approach to personality: affective neuroscience personality theory (ANPT) (Davis et al., 2003; Panksepp, 1998). The ANPT has utilized the Affective Neuroscience Personality Scale (ANPS), designed to measure subcortical affective systems: care, play, seek, anger, sadness, and fear (Davis et al., 2003). The account suggests that the strengths and weaknesses found in the universally shared subcortical affective systems are the epigenetic predictors of personality traits (Davis and Panksepp, 2011, 2018; Panksepp and Biven, 2012). These systems are shaped by nurture, namely early life experiences and learning (Panksepp, 1998). Thus, ANPT initiated a paradigm shift toward a holistic approach to personality, integrating nature and nurture (Montag and Panksepp, 2017).

Despite this shift, cultural influences on ANP profiles were still unexplored. Cross-cultural Affective Neuroscience (CAN, Özkarar-Gradwohl, 2012) aimed to measure the influence of culture on these basic affective systems. CAN now constitutes a substantial body of work (Özkarar-Gradwohl et al., 2014; Özkarar-Gradwohl, 2019; Özkarar-Gradwohl et al., 2018; Özkarar-Gradwohl and Turnbull, 2021), including a collaborative meta-analysis (Marengo et al., 2021). Its hypothesis is that universal subcortical affective systems (which are epigenetic predictors of personality) are regulated (reinforced and/or inhibited) differently across cultures, via varying parenting styles, family models, emotion socialization, and cultural norms (Özkarar-Gradwohl, 2019). Therefore, CAN predicts both universal similarities and cultural differences in cross-cultural ANPS comparisons. As a hybrid model (Irish, 2025), CAN seeks to integrate not only nature and nurture, but also universal and cultural findings on emotion.

Can culture and universalism co-exist in affective neuroscience? This opinion paper evaluates the main CAN hypothesis by categorizing its evidence as quasi-universal similarities and cultural differences. It observes improvements in methodology for analyzing connectedness and separateness and suggests further investigation of the interaction of culture and gender. Finally, limitations and recommendations are discussed.

## Quasi-universal similarities

A route to assess the universalism comes from the standardization studies of the ANPS in different cultures. Thus far, the ANPS has been translated to Spanish, French, Turkish, Norvegian, Italian, Polish, Portuguese, Persian, Japanese, Chinese, German, Serbian, Brazilian Portuguese, Russian (Abella et al., 2011; Amiri and Azad-Marzabadi, 2017; Cwojdzińska and Rybakowski, 2015; De Almeida, 2016; Geir et al., 2014; Gurfinkel et al., 2018; Montag et al., 2017; Narita et al., 2017; Özkarar-Gradwohl et al., 2014; Pahlavan et al., 2008; Pascazio et al., 2015; Pingault et al., 2012; Reuter et al., 2017; Sindermann et al., 2018; Volf and Privodnova, personal communication)^1^ and standardized for Canada and Hong Kong (Orri et al., 2016; Yu, 2016;).

The original reliability and validity findings (Davis et al., 2003) were confirmed by all these studies, though a systematic review was not reported. The reliability of the ANPS, across different cultures, was first reviewed by CAN (Özkarar-Gradwohl, 2019). The original U.S. study had shown positive intercorrelations within positive and negative affects, suggesting higher-order universal personality factors (Davis et al., 2003). CAN pointed out that this finding also exists for Spanish, French, Turkish, Italian, Japanese, and German samples (Özkarar-Gradwohl, 2019).

The *construct validity* of the original ANPS study (Davis et al., 2003) was measured in relation to the Big Five Scales (B5S) (Goldberg, 1990). CAN detected similar correlation patterns in Spain, Germany, Japan, and Türkiye (Özkarar-Gradwohl, 2019). These universal patterns included positive correlations between Fear/Sadness/Anger and neuroticism, high Care/low Anger and agreeableness, Seek, and openness to experience, and finally between Play and Extraversion. However, certain culturally specific correlational patterns between the ANPS and B5S were also observed. Notably, Play was positively correlated with Agreeableness in the Japanese and Turkish samples, Seek was positively correlated with all B5 (except Neuroticism), and Fear was negatively correlated with Extraversion in the Japanese sample (Narita et al., 2017; Özkarar-Gradwohl et al., 2014). Therefore, universal patterns need to be investigated further and more systematically.

Marengo et al. (2021) conducted a meta-analysis of all studies that included the ANPS-B5S findings (21 samples from 12 countries). Significant relationships were obtained between Fear/Sadness/Anger and Neuroticism; Seeking and Openness; Play and Extraversion; and high Care/low Anger and high Agreeableness. Therefore, this first meta-analysis showed that the cortical cognitive Big 5 factors can be universally mapped onto the subcortical affective roots measured by the ANPS.

Moreover, a CAN review of gender effects in 15 countries showed that universal gender effects do exist: notably higher Care and Sadness for females, and equal levels of Seeking and Anger for both genders (Özkarar-Gradwohl and Turnbull, 2021). In sum, *the universalism of subcortical affective traits has been well established*: higher order personality factors, gender effects, links between the affective personality traits and the cognitive B5 traits, and gender effects on ANPS. However, clarification is needed about where universalism interacts with culture.

## Cultural differences

Cultural psychology emerged with an initial focus on universalism and a late interest in cultural differences (Chiao and Ambady, 2007; Irish, 2025; Khan et al., 2017; Matsumoto and Wilson, 2022; Thalmayer et al., 2022). Cultural neuroscience also warned that almost 90% of fMRI publications come from Western countries, lacking diversity (Chiao, 2017; Chiao and Blizinsky, 2010). Similarly, the influence of culture (and gender) on personality was previously investigated for B5 (Schmitt et al., 2007, 2008), but not yet for the ANPS. CAN was the first to address this issue (Özkarar-Gradwohl, 2012) by demonstrating the presence of cultural differences, besides universal similarities (Özkarar-Gradwohl et al., 2014, 2018; Özkarar-Gradwohl, 2019; Özkarar-Gradwohl and Turnbull, 2021).

The first CAN research between the U.S. and Türkiye (Özkarar-Gradwohl et al., 2014) and the first Euro-Asian CAN research between Japan, Türkiye, and Germany (Özkarar-Gradwohl et al., 2018) reported significant differences among cultures. These differences were elaborated as reflections of cultural norms, family models, mothering/parenting styles, and emotional socialization in each culture. Themes included: Western individualism and hedonism in reinforcing positive affects, Eastern collectivism and holistic philosophy in inhibiting Anger and normalizing Sadness; and German rationalism in suppressing most affects (Özkarar-Gradwohl et al., 2014, 2018). Other themes were separation-reinforcing parenting styles in the West vs. the symbiotic parenting styles in the East (Friedlmeier and Trommsdorff, 1998; Friedlmeier et al., 2011; Roland, 1996) and the separation-without-detachment style in Türkiye (Özkarar-Gradwohl et al., 2014, 2018). However, these discussions were mostly theoretical assumptions, and additional methods are needed to test them further. Moreover, the influence of all types of caregiving styles, including fathering styles, needs to be explored.

Moreover, these initial CAN studies were carried out only between two or three cultures (Özkarar-Gradwohl, 2019); therefore, a large-scale ANPS review, on gender effects, was completed across 15 countries (Özkarar-Gradwohl and Turnbull, 2021). Strikingly, Fear (and to some extent Play) showed geographic variability. Western females had higher Fear than their males, while Eastern females did not differ from males. Discussions were again theoretically assumptive, such as the influences of individualism/collectivism on oxytocin and serotonin (Chiao and Blizinsky, 2010; Luo and Han, 2014). Nevertheless, these data again suggested cultural differences.

Cultural differences were also observed in affective polarization and affective complexity (Özkarar-Gradwohl, 2019). Although positive and negative affects are universal higher-order personality factors, certain cultures showed more intercorrelations between positive and negative affects (e.g., Persian, Turkish, and Japanese samples). This was aligned with the literature showing increased affective complexity and dialectical thinking in Eastern cultures and affective polarization in Western cultures (Peng and Nisbett, 2000; Spencer-Rodgers et al., 2010).

In sum, CAN demonstrated evidence for *cultural differences in subcortical affective traits, as measured by ANPS*. These supplied primary examples of where the universalism of ANPT can interact with culture. Moreover, to improve the discussions beyond the theoretical assumptions, these culture-specific findings were later explored in relation to connectedness/separateness.

## Connectedness and separateness

Cultural studies define connectedness (interdependence/relatedness) and separateness (independence/autonomy) as two distinct ways of constructing the self in relation to the social group (Markus and Kitayama, 2010; Singelis, 1994). The two main variables influencing connectedness and separateness are found to be culture and gender, with Eastern cultures (and females) having higher connectedness, while Western cultures (and males) have higher separateness (Bekker et al., 2011; Kagitçibaşi, 2005; Markus and Kitayama, 2010; Singelis, 1994; Triandis, 1995; Van Assen and Bekker, 2009). However, *as regards culture*, most studies defined their samples simply by geographical location (East vs. West) (e.g., Hofstede and McCrae, 2004; Triandis and Suh, 2002). These geographical stereotypes need to be corrected by objective assessments of connectedness/separateness (for criticisms see, Kirmayer, 2012; Voronov and Singer, 2002).

Therefore, CAN integrated the assessment of interdependent and independent self-construals (Özkarar-Gradwohl et al., 2018). While interdependent self-construals (connectedness) decreased from East to West (from Japan to Türkiye to Germany), independent self-construals (separateness) did not show a gradual westward increase. Thus, widely accepted German individualism appears not to be based on higher separateness but on lower connectedness. Clearly, cultures must not be identified simply as collectivistic or individualistic based on geography (Özkarar-Gradwohl, 2019).

The loadings of ANPS and B5S on self-construals also varied across cultures (Özkarar-Gradwohl et al., 2018). While both ANPS and B5S loaded on self-construals in Japan and Türkiye, only B5S did so in Germany. For *independency*, the common positive B5 predictor was Extraversion for all samples, while the underlying effect varied across cultures. For *interdependency*, the common negative B5 predictor was Openness to Experience, while other affective/cognitive predictors varied. Thus, different emotions and cognitions seemed to trigger connectedness and separateness in different cultures (Özkarar-Gradwohl et al., 2018; Özkarar-Gradwohl, 2019).

As regards gender, the finding (Özkarar-Gradwohl and Turnbull, 2021) of high Care and Sadness in females aligns with the suggestions that females show more attachment (connectedness) and separation distress (Panksepp, 1998), as well as higher relatedness (e.g., Fişek, 2009; Van Assen and Bekker, 2009). However, gender differences on B5 (Schmitt et al., 2017) and on ANPS (Özkarar-Gradwohl and Turnbull, 2021) increase when moving from East to West. As the quasi-universal finding for higher Care in females was not found in China and Japan, CAN discussed whether collectivism (connectedness) and individualism (separateness) might influence the gender effects on Care (which requires connectedness) differently.

Another striking gender effect was for Fear. While most females in North America and Europe had higher Fear than males, no such findings were found among Asian females (Özkarar-Gradwohl and Turnbull, 2021). The Eastern philosophy of connectedness might have a preventive influence on separation anxiety, which seems to be higher in autonomy-reinforcing individualistic cultures (De Vaus et al., 2017). This assumption is supported by neurogenetic findings that serotonergic and oxytocinergic systems (thus, mood and anxiety) appear to be mediated by collectivism (Chiao and Blizinsky, 2010; Luo and Han 2014). Interestingly, the levels of Anger (an affect disrupting connectedness/ activating separateness) did not differ between genders (Özkarar-Gradwohl and Turnbull, 2021) but between cultures (Özkarar-Gradwohl et al., 2014, 2018), pointing to the need for further cultural studies on testosterone.

Clearly, future CAN studies must investigate the interaction effects of culture and gender on ANPS.

## Discussion

The limitations of all cultural sciences also apply to CAN. Notable examples are the narrowness of the sampling range (e.g., limitations in age and education, comparisons between urban/rural or ethnic groups) and of procedures (e.g., defining samples based on geography and observing variables loaded on raw scores). There may also be a question of measurement invariance for ANPS. However, the meta-analysis by Marengo et al. (2021)) shows that ANPS possess strong measurement invariance not only across genders and over time but also across cultures and forms of ANPS. Taking these into consideration, CAN demonstrates sufficiently that universally shared basic affective systems are regulated differently across cultures. CAN thus presents a hybrid model where universal similarities and cultural differences can co-exist (Irish, 2025) and where each culture has a different (perhaps unique) affective personality profile (Özkarar-Gradwohl, 2019). However, methodological issues still need improvement, and discussions need to be grounded beyond assumptions.

A systematic improvement would be establishing a global affective map (designed such as heat maps for each basic affect, separately for both genders) and grounding this affective map. Such a map may be developed by standardizing the scores from different versions of the ANPS and comparing these standardized z-scores. The outcome might enlighten the global affective networks alongside the regional similarities. It may also facilitate the observation of both between- and within-culture variations. On a macro-scale, such a map may help the cross-cultural arguments evolve beyond simple East-West dichotomies by also incorporating North-South comparisons. The validation of this map can be supplied by its correlations to the B5 (Schmitt et al., 2007, 2008) and Self-construals (Vignoles et al., 2016).

To ground the global affective map and the discussions, CAN needs to be integrated into surrounding research fields. First, the ANPS findings should be combined with developmental nurture variables, such as duration of breastfeeding, onset of toilet training, co-sleeping in parents' rooms, maternal and paternal attachment styles, family models, and emotional socialization. Such developmental grounding might support the main premises of CAN by enlightening the influence of nurture on basic affective systems. Second, neuroscientific variables might be better integrated into CAN studies. These might include neuro-anotomical, neuro-functional, and neuro-chemical assessments to better clarify the ‘nature' variables for CAN. In sum, grounding the global affective map in these developmental and neuroscientific methods would clearly enhance CAN.

The evidence in this paper supports the main hypothesis of CAN, that universal affective systems are also regulated differently by cultures. Thus, universalism and culture *can* co-exist in affective neuroscience. Applying the recommendations suggested above may strengthen this newborn theory. Notably, these findings can also be used to develop culture-sensitive therapies, where psychotherapy techniques and goals are modified according to cultural contexts (Özkarar-Gradwohl et al., 2018).

## Footnotes

1 Volf, N. V., and Privodnova, E. Y. (personal communication). *The Russian Standardization of the ANPS*. State Scientific-Research Institute of Physiology and Basic Medicine, Lab. of Cognitive Physiology: Novosibirsk, Russia.
